# Zebrafish as a model for crystallin-associated congenital cataracts in humans

**DOI:** 10.3389/fcell.2025.1552988

**Published:** 2025-03-26

**Authors:** Jennifer L. Rossen, Antionette L. Williams, Brenda L. Bohnsack

**Affiliations:** ^1^ Division of Ophthalmology, Ann and Robert H. Lurie Children’s Hospital of Chicago , Chicago, IL, United States; ^2^ Department of Ophthalmology, Northwestern University Feinberg School of Medicine, Chicago, IL, United States

**Keywords:** congenital cataracts, zebrafish, crystallins, cryaa, cryab

## Abstract

Congenital cataracts are a leading cause of vision loss in children and can be an isolated finding or associated with systemic abnormalities. Isolated congenital cataracts are most commonly associated with pathogenic variants in one of the Crystallin genes. The α-Crystallins are small heat shock proteins that act as chaperones in the lens and other organs throughout the body to prevent protein aggregation and maintain tissue function. In contrast, the ß- and γ-Crystallins are structural proteins that are predominantly expressed in the mature lens and regulate its refractive index. However, the role of the Crystallins during lens development such that pathogenic variants result in inherited cataracts is less well-defined. As zebrafish allow real-time visualization of lens development, genetic manipulation of both the endogenous Crystallin genes as well as the use of transgenic overexpression of identified pathogenic variants yields important insight into the pathogenesis of congenital cataracts. Herein, we review the similarities and differences between human and zebrafish Crystallin genes. Further, we discuss the use of zebrafish as a model for congenital cataracts and explore the mechanisms that underlie the role of Crystallins in lens development. A better understanding of the genetic causes of congenital cataracts will lead to breakthroughs in preventing blindness from congenital cataracts and associated complications.

## Introduction

Congenital lens disorders and cataracts are estimated to affect 20,000 to 40,000 children worldwide (Rahi et al., 2001; Sheeladevi et al., 2016; Bell et al., 2020). A challenge with congenital lens disorders is that there are multiple etiologies (e.g., genetic, infectious, toxic), and the eye findings can be found in isolation (∼70%), associated with other ocular anomalies (∼15%), or as part of wider systemic syndromes (∼15%) (Bell et al., 2020; Yi et al., 2011; Patel et al., 2019). Isolated unilateral ocular findings, especially without family history of similar eye findings, are typically attributed to somatic mutations, however it is important to still consider genetic work-up in these patients as inherited eye diseases can often present asymmetrically due to incomplete penetrance, mosaicism, epigenetic or environmental factors. Patients with bilateral findings should be referred for genetic evaluation in order to determine if there is an identifiable genetic etiology and potential systemic involvement (Bell et al., 2020; Gillespie et al., 2016).

## Crystallin and inherited cataracts in humans

Variants in the crystallin genes (*CRY*) are predicted to account for approximately half of isolated non-syndromic inherited cataracts, however, their role in lens development is not well-defined (Shiels and Hejtmancik, 2015; Shiels and Hejtmancik, 2017; Berry et al., 2020). The crystallins comprise ∼90% of the proteins within the mature lens and are divided into α, ß, and γ subtypes, all of which have been associated with autosomal dominant and/or autosomal recessive congenital cataracts (Bell et al., 2020; Berry et al., 2020; Wistow, 2012). The most commonly reported congenital cataract-causing variants involve the *CRYAA*, *CRYGD*, *CRYBB1*, *CRYBA1* genes (Berry et al., 2020; Wistow, 2012).

In humans there are 2 α-Crystallin subunits, the αA and the αB, which are encoded by the *CRYAA* and the *CRYAB* genes (Berry et al., 2020; Wistow and Piatigorsky, 1988; Horwitz, 2003). The α-Crystallins belong to the small heat shock protein superfamily. They serve as molecular chaperones preventing other proteins from aggregating, thereby maintaining lens clarity (Wistow, 2012; Wistow and Piatigorsky, 1988). Further, these proteins exist in oligomers that are often greater than 20 subunits and have a highly conserved crystallin domain, hydrophobic N-terminal and flexible C-terminal extension (Wistow, 2012). The crystallin domain responds to environmental changes (e.g., pH and metal ions) and together with the N-terminal domain bind destabilized proteins. These oligomers are in a constant state of flux as the exchange of smaller multimers is necessary for chaperone function (Wistow, 2012). Further, *CRYAB* evolved as a stress-induced heat shock protein and is modified by phosphorylation resulting in changes in oligomer size and rate of protein binding (Wistow, 2012; Clark et al., 2012; Thornell and Aquilina, 2015; Muranova et al., 2018). While *CRYAA* is predominantly expressed within the lens, *CRYAB* is also found in other tissues including cardiac and skeletal muscles. Thus *CRYAB* variants in addition to congenital cataracts is associated with cardiomyopathies and skeletal myopathies (Berry et al., 2020; Reis and Semina, 2019).

The ß- and γ-Crystallins are a separate family of proteins which are predominantly expressed in the lens and function to maintain its refractive index (Wistow, 2012; Slingsby and Clout, 1999). In humans, the ß-Crystallins consist of six subunits encoded by the *CRYBA1*, *CRYBA2*, *CRYBA4*, *CRYBB1*, *CRYBB2*, and *CRYBB3* genes, with the first three being acidic forms and the last three basic (Berry et al., 2020; Wistow, 2012). The 5 γ-Crystallins are encoded by the *CRYGA*, *CRYGB*, *CRYGC*, *CRYGD*, and *CRYGS* genes and are clustered on Chromosome 2q.9 (Wistow, 2012; Shiloh et al., 1986), Other mammals have three additional γ-Crystallin genes (*CRYGE*, *CRYGF*, and *CRYGN*), however, these have become pseudogenes in humans with no evidence of expression (Wistow, 2012; Riazuddin et al., 2005; Wistow et al., 2005). The ß- and γ-Crystallins have a different structure than the α-Crystallins, consisting of two domains each composed of Greek key motifs (Berry et al., 2020; Wistow, 2012). ß-Crystallins also can have N- or C-terminal extensions which allows formation of small oligomers, while γ-Crystallins are found as simple monomers (Wistow, 2012; Wistow and Piatigorsky, 1988).

## Zebrafish as a model for understanding lens development

Zebrafish are important in modeling and investigating congenital diseases due to the genetic and anatomic similarities with the human eye and the accessibility of real-time imaging (Gross and Perkins, 2008). The live imaging is particularly useful for visualizing subtle changes in the lens due to genetic variants that could otherwise be missed by *in vitro* studies (Greiling and Clark, 2009; Mochizuki et al., 2017). In the past, numerous advances in lens embryology were made using chick lenses. However, the zebrafish is likely a better model as its embryology more closely resembles the human lens. The advent of techniques, such as CRISPR-Cas9 gene manipulation and Cre-Lox tissue and time specific knockouts, give important insight into eye development and the evaluation of genetic ocular anomalies including inherited cataracts (Zhao et al., 2021; Peng et al., 2024).

Lens development in zebrafish is similar to that in humans and is closely linked to overall eye development which involves interactions between neural epithelium, surface epithelium and neural crest cells. Evagination of the neural epithelial-derived optic vesicles from the diencephalon of the developing brain occurs at the six-somite stage (12 h post-fertilization (hpf)) in zebrafish and around day 25 in humans (Chow and Lang, 2001; Zagozewski et al., 2014; Abitbol, 2015). The optic vesicles then interact with the overlying surface epithelium to stimulate invagination of the bilayered optic cup and to induce the lens placode within the surface ectoderm in zebrafish by 16 hpf and in humans around day 28 (Canto-Soler and Adler, 2006; Fuhrmann, 2010). As the optic cup differentiates into the outer retinal pigment epithelium and the inner neural retina, the lens vesicle separates from the overlying surface epithelium which will ultimately become the corneal epithelium (Fuhrmann, 2010; Cardozo et al., 2023). While in humans a hollow lens vesicle forms, in zebrafish the lens cells delaminate en masse from the surface ectoderm (Dahm et al., 2007). This difference may be due to the close apposition of the lens and the cornea in the zebrafish eye that creates a singular optical refracting surface in an aqueous habitat *versus* the dual refracting surfaces of the cornea/tear film and the lens in the human eye exposed to air. Nevertheless, this embryological difference does not appear to affect the transparency of the lens or refractive power, and is not likely to be influenced by crystallin function or their variants. Concurrently, the mesodermal- and neural crest-derived periocular mesenchyme migrates into the anterior segment both between the edge of the optic cup and the surface ectoderm as well as through the optic fissure along the inferonasal aspect of the cup (Williams et al., 2017; Williams and Bohnsack, 2020). Within the anterior segment, the neural crest cells then migrate and contribute to the corneal stroma and endothelium, iris stroma, ciliary body stroma, and sclera (McMenamin, 1989). The optic fissure also transmits the hyaloid vasculature that is the main vascular network inside the vitreous cavity and connects with the tunica vasculosa which encases the lens and supports the developing anterior chamber (Lingam et al., 2021). Both the hyaloid vasculature and the tunica vasculosa resorb following anterior segment development, and failure to do so can result in mild abnormalities such as Mittendorf dots, Bergmeisters papillae, anterior polar cataract, and pupillary membranes or severe visually significant anomalies such as anterior and/or posterior persistent fetal vasculature (Shastry, 2009; Lutty and McLeod, 2018).

Within the lens, the posterior lens epithelial cells differentiate into primary lens fiber cells (Cvekl and Ashery-Padan, 2014; Kumar and Reilly, 2020). These cells proliferate to fill the primordial lenticular space and remain within the center of the lens to form the fetal lens nucleus. Daughter cells continue to proliferate and give rise to lens epithelial cells and lens fiber cells, which are evident in the zebrafish by 36 hpf and in human embryos by 60 days (Cvekl and Ashery-Padan, 2014). Lens epithelial cells are a cuboidal monolayer and secrete a basement membrane which clinically is referred to as the lens capsule. The lens capsule protects the lens fibers and facilitates through the zonules the attachment of the lens to the circumferential ciliary processes (Cvekl and Ashery-Padan, 2014; Kumar and Reilly, 2020).

The majority of cells become secondary lens fiber cells in the meridional zone which can be seen in the 72hpf zebrafish larvae. These cells exit the cell cycle and elongate to form concentric shells throughout life that give rise to the lens nucleus and surrounding cortex. Differentiation into lens fiber cells involves downregulation of epithelial markers, increase in the synthesis and orderly packing of crystallin proteins, increase in expression of water channels such as aquaporins, and degradation of cytoplasmic organelles in order to provide the refractive power of the lens and maintain its transparency (Bassnett, 2009; Bassnett et al., 2011). Various transcription factors, *c-maf*, *l-maf*, *prox1*, and *pax6* have been shown in animal models to be essential in regulating the differentiation of lens epithelial cells into fiber cells and in chick and mice specifically the expression of crystallin gene expression (Cvekl and Ashery-Padan, 2014; Yang et al., 2006). Further, since the lens fiber cells lack organelles, protein turnover is regulated by the chaperone activity of the α-Crystallin proteins (Sharma and Santhoshkumar, 2009). On the other hand, the ß- and γ-Crystallins regulate the refractive index of the lens as the fibers continue to deposit post-natally (Wistow, 2012; Sharma and Santhoshkumar, 2009). Thus, the lens is a dynamic tissue and understanding its early development and further growth is important in appreciating disease pathogenesis.

Cataract formation occurs in humans as well as zebrafish with age, due to aggregation of proteins resulting in loss of lens clarity (Khatiwada et al., 2024). Conversely, congenital cataracts are a result of disruption in lens development and have been attributed to numerous genes including those that encode transcription factors that regulate signaling pathways, cytoskeletal proteins that serve as intracellular scaffolds, and membrane-bound proteins that regulate cell-cell communication within the avascular lens (Bell et al., 2020; Berry et al., 2020). Interestingly, although variants in crystallin genes are the most frequently reported gene family associated with congenital cataracts, their role in lens development is less well-defined, as they are most commonly thought of as maintaining tissue clarity in the mature lens.

## Crystallin genes in zebrafish

While crystallin proteins are important in maintaining lens clarity and refractive index post-natally, the *CRY* genes also play a role during lens development. Animal studies are just starting to give insight into the signaling networks that are regulated by the different *cry* genes. Zebrafish underwent ancestral teleost genome duplication during evolution resulting in multiple genes (sometimes two to four separate genes) for many mammalian genes (Taylor et al., 2003). As a result, while humans have 2 *CRYA*, 6 *CRYB* and 5 *CRYG* genes, zebrafish have 3 *crya*, 13 *cryb*, and 40 *cryg* genes. In addition, there are four *crybg* genes, which are predominantly non-lens paralogs (Farnsworth et al., 2021). There is a high degree of homology between zebrafish and human α-Crystallins, but more divergence with the ß and γ proteins (Farnsworth et al., 2021; Posner, 2003). Other than gene expression data, there is limited functional data available on either the ß-and γ-Crystallins (Wistow et al., 2005; Farnsworth et al., 2021). Further, zebrafish lack the human equivalents of *CRYGA*, *CRYGB*, *CRYGC*, and *CRYGD* genes as the majority of *cryg* genes in fish belong to the aquatic specific γM group. The γM group are methionine rich, affording higher density proteins that are hypothesized to facilitate light refraction in aqueous rather than atmospheric environments (Mahler et al., 2013). Within the adult zebrafish lens, the γ-Crystallins are most prevalent (47%) with the ß-Crystallins second at 36% of the total protein. Only a small portion of proteins are α-Crystallins (8%) in the mature lens (Posner et al., 2008). Nevertheless, the smaller prevalence of the α-Crystallins in the post-natal lens does not preclude their importance in lens development.

### α-crystallins

The zebrafish Cryaa protein shares 72% identity in amino acid sequence with the human CRYAA protein (Table 1) (Runkle et al., 2002) Functional analysis shows similar chaperone activity between the zebrafish Cryaa and human CRYAA proteins. However, the zebrafish form is less stable at high temperatures, and functions at lower temperatures compared to the human, which likely reflects species-specific environmental differences (Dahlman et al., 2005). However, there are two *cryab* genes, *cryaba* and *cryabb*, due to ancestral teleost genome duplication (Smith et al., 2006; Mishra et al., 2018). The Cryaba and Cryabb proteins share 61% and 58% homology, respectively to the human CRYAB protein (Table 2) (Smith et al., 2006) The zebrafish αB-crystallins overall show less baseline chaperone activity than the αA-Crystallins, which may correlate with its function as a stress-induced heat shock protein that requires post-translational modifications such as phosphorylation to become fully active (Smith et al., 2006). Single cell transcriptome analysis recently demonstrated that *cryaa* is exclusively expressed within the lens fiber cells and is the earliest expressed Crystallin gene, starting by 48 hpf and increasing in expression over the next 3 days. In contrast *cryaba* and *cryabb* genes are predominantly expressed in non-ocular tissues during the zebrafish embryonic and (0–72 hpf) early larval (72–120 hpf) phases (Farnsworth et al., 2021).

**TABLE 1:** Human vs zebrafish CRYAA comparison.

	Human *CRYAA*	Zebrafish *cryaa*
Location	Chromosome 21 21:43.17–21	Chromosome 1 29,096,881–29,100,923
Transcript length	1,139 bases	1,417 bases
Protein length	173 amino acids	173 amino acids 72% homology
Embryonic expression	Lens epithelial and fiber cells	Lens epithelial and fiber cells
Chaperone activity	Lower chaperone activity at lower temperatures than zebrafish cryaa	Lower thermal stability at high temperatures compared to human CRYAA

**TABLE 2:** Human vs zebrafish CRYAB comparison.

	Human *CRYAB*	Zebrafish *cryaba*	Zebrafish *cryabb*
Location	Chromosome 11 11.q.23.1	Chromosome 15 18,119,618–18,124,678	Chromosome 5 57,641,554–57,650,998
Transcript length	964 bases	1,335 bases	2,192 bases
Protein length	175 amino acids	168 amino acids 61% homology	180 amino acids 58% homology
Embryonic expression	Lens epithelial/fiber cells	Lens epithelial/fiber cells	Lens epithelial/fiber cells
	Brain		Brain
	Heart		Heart
	Skeletal muscle		Skeletal muscle
	Liver		Liver
Chaperone activity	More similar to Cryabb activity	Lower chaperone activity compared to Cryabb	Greater chaperone activity compared to Cryaba

### Cryaa

Attention on the α-Crystallins as causes of congenital cataracts in zebrafish was first brought into focus due to the spontaneous *cloche* mutant, which in addition to hematopoietic and vascular abnormalities was also found to have cataracts by 72 hpf (Goishi et al., 2006). Initial studies reported decreased *cryaa* mRNA and Cryaa protein expression, however later studies with more sophisticated molecular capacity demonstrated that the cataracts were not due to lack of *cryaa*, but rather from general disruption of lens cell homeostasis (Goishi et al., 2006; Posner et al., 2019). Nevertheless, Posner et al. showed that c*ryaa* mutant zebrafish created by CRISPR-Cas9 gene editing had lens abnormalities by 72 hpf (Posner et al., 2023). Approximately 60% of *cryaa −/−* mutant embryos obtained from *cryaa −/−* x *cryaa −/−* crosses showed lens changes that had varying levels of severity ranging from subtle areas of “roughness” in the primary fiber cells or along the peripheral fiber cell boundaries to more severe findings of central pitting and abnormal interfaces between fiber cells (Posner et al., 2023). Further, there was a mild delay in lens fiber cell denucleation from 72 to 96 hpf, however, ultimately loss of *cryaa* did not prevent loss of cell nuclei within the fiber cells. Zou et al. also reported a *cryaa* −/− mutant zebrafish that was created by TALEN nuclease gene editing and showed similar phenotype and penetrance (∼50%) amongst progeny derived from heterozygous *cryaa* ± x *cryaa* ± crosses (Zou et al., 2015). Interestingly, they found that embryos derived from homozygous *cryaa −/−* x *cryaa −/−* crosses showed higher phenotypic penetrance of greater than 90% and attributed this difference to *cryaa* transcripts received through maternal transmission in the heterozygous crosses. As the mutant analysis performed by Posner et al. were also derived from homozygous *cryaa −/−* crosses, the difference in phenotypic penetrance is not clear. A possible explanation could be the influence of the wildtype background that was utilized. Interestingly, the AB strain (which was used in Zou et al.) shows higher frequency (16%) of cataracts at 96 hpf compared to the TL strain (9%) (Khatiwada et al., 2024). The studies by Posner et al. utilized the ZDR strain, which to date has no published data as to the frequency of early onset cataracts, but 27% of this wildtype strain develop cataracts by 18 months of age (Posner et al., 2024; Posner et al., 2023). Due to differences in the propensity for baseline cataracts in different strains, the type of strain used in a study should be taken into consideration.

Additional animal studies on the function of the *cryaa* gene have utilized pathogenic variants identified in patients with congenital cataracts. The R49C variant was first identified in a 4-generation family with inherited cataracts and alters the N-terminal domain of the CRYAA protein (Mackay et al., 2003). This variant is predicted to have increased affinity for undamaged proteins thereby saturating the binding sites, leading to chaperone/protein aggregation and ultimately cataract formation (Koteiche and McHaourab, 2006; McHaourab et al., 2009). Zebrafish which were knock-ins of the R49C variant, created by injection of the transgene under a lens-specific promotor showed a similar phenotype to the *cryaa −/−* mutants with lens changes detectable by 72 hpf in many, but not all affected fish (Mishra et al., 2018; Wu et al., 2018). The frequency of lens changes increased when the R49C *cryaa* transgene was paired with the *cryaa −/−* null background. Interestingly, the R49C variant caused aggregation of destabilized crystallin proteins resulting in more frequent lens abnormalities in zebrafish larvae (Wu et al., 2018). Knock-in of the R49C variant in mice showed cataracts by 2 days post-natally, and further studies demonstrated various alterations within the lens including altered biosynthesis and metabolism pathways. This led to increased creatine kinase activity and lactic acid and changes in histone expression, specifically increased H2 and decreased H3 (Andley et al., 2018; Frankfater et al., 2020). Additional studies in mice have shown that disruption of various H2 and H3 genes also results in lens abnormalities through changes in DNA methylation and ultimately downstream gene expression (Vetrivel et al., 2019; Andley et al., 2020; Hamilton et al., 2020). Similar molecular studies on the effects of the R49C variant as well as downstream targets of *cryaa* during lens development have yet to be performed in zebrafish. In summary, *cryaa* plays a role in zebrafish lens development such that alterations in expression increase risk of congenital lens abnormalities, but additional studies are required to understand the downstream targets of *cryaa* in the zebrafish lens.

### Cryab

Although *cryaa* variants are more commonly associated with congenital cataracts, *cryab* variants have also been identified in patients with inherited cataracts. In zebrafish, there are differing reports regarding *cryaba −/−* and *cryabb* −/− null mutants. Posner et al. reported that *cryaba −/−* and the *cryaba −/−* larvae showed no significant developmental lens abnormalities at 72 and 96 hpf (Posner et al., 2023). However, this same group later showed that close to half of *cryaba* −/− mutant zebrafish developed age-related cataracts by 2 years of age, compared to approximately 25% in wildtype and *cryaa* −/− mutant zebrafish (Posner et al., 2024). In contrast, Mishra et al. found cataracts in 72–96 hpf in *cryaba* ± and *−/−* as well as *cryabb* ± and *−/−* zebrafish, with higher frequencies seen in homozygous mutants compared to heterozygous fish. Further, the double *cryaba −/−; cryabb* −/− mutants and the double *cryaa* −/−; *cryaba* −/− mutants showed even higher frequency of lens abnormalities, ranging between 75% and 95% of fish (Mishra et al., 2018). The *cryaba* −/− and *cryabb −/−* mutant fish in Mishra et al. did have a more mild phenotype compared to the morpholino oligonucleotide knockdown of Cryaba and Cryabb expression through inhibition of protein translation (Zou et al., 2015). However, this may be due to systemic toxic effects and inhibition of overall growth and development from the morpholinos. This difference in phenotypes between the Posner and Mishra studies may be due to the pathogenicity of the knockout, although both studies verified absence of expression of the corresponding mRNA. Further, lenses from adult *cryabb −/−* mutants in Posner et al. were found to express a truncated version of the Cryabb protein which is predicted to contain the anti-aggregation chaperone site. The presence of this truncated form of Cryabb was not verified in embryonic lenses, as well as this does not account for the difference in phenotypes between the two c*ryaba −/−* mutants presented by Posner et al. and Mishra et al (Posner et al., 2023) Similar to as discussed above, the differences in phenotypes may also be due to the strain background, but additional studies are required to further understand this discrepancy.

Like the *CRYAA* gene, certain pathogenic variants have been identified in *CRYAB* in association with inherited cataracts, and this information has been utilized to study the effects of these variants on protein structure and function and the subsequent downstream effects within tissues (Vicart et al., 1998). A R120G variant of *CRYAB*, was found in affected members of a family to be associated with cardiomyopathy, skeletal myopathy, and “discrete lens opacities” of unknown age at onset (Vicart et al., 1998). The R120G variant in *CRYAB* is located within the α-crystalline domain and results in protein destabilization with oligomer disruption and subsequent aggregation (Bova et al., 1999; Darvazi et al., 2024). The effects of the R120G variant on the CRYAB protein do not seem to have the same extent of effects as the R49C variant has on the CRYAA protein (Wu et al., 2018), and their phenotypes in humans appear to vary, as well (Berry et al., 2020; Mackay et al., 2003). In zebrafish, knock-in of a R120G *cryab* transgene under a lens-specific promotor resulted in minor lens defects, which were magnified in penetrance and severity when crossed into the *cryaba −/−; cryabb −/−* double mutants (Wu et al., 2018). Mouse studies also found that knocking in the R120G *Cryab* variant caused cataracts, but with a slightly delayed onset (at 2 weeks) compared to the R49C *Cryaa* variant (Andley et al., 2018).

In mice, the R120G *Cryab* variant had less effect on histone expression, but did alter metabolic pathways involved in nucleic acid synthesis and degradation and endoplasmic reticulum protein processing (Andley et al., 2018; Frankfater et al., 2020). Studies assessing downstream effects of the R120G *cryab* variant in zebrafish are lacking. Nevertheless, these studies give important insight into the role of *cryaba* and *cryabb* in lens development and the effects of pathogenic variants that result in early cataract formation.

Additional studies link together the αB-crystallins with regulating lysosomal activities, which are necessary for organelle degradation within differentiating lens fiber cells (Costello et al., 2013; Morishita et al., 2013; Chauss et al., 2014; Cui et al., 2020). Pathogenic variants in the *HSF4* gene, which encodes the heat shock transcription factor, HSF4, are associated with cataracts in humans and similarly knockout of *Hsf4* in mice or zebrafish results in early onset cataracts (Bu et al., 2002; Min et al., 2004; Ke et al., 2006; Gao et al., 2017). Further studies demonstrated decreased expression of *cryaba* and aberrantly increased lysosomal pH in the lenses of *hsf4 −/−* mutant fish (Cui et al., 2020). This *in vivo* zebrafish data paired with *in vitro* studies from mouse lens epithelial cells suggests that Hsf4 regulates expression of αB-crystallin, which binds to the ATP6V1A-mTORC1 complex. As αB-crystallin stabilizes the complex and prevents degradation, the ATP6V1A as part of the proton pump maintains the acidic environment within the lysosome while mTORC1 is activated on the surface of the lysosome (Cui et al., 2020). Within zebrafish, knockout of *hsf4* is hypothesized to lead to decreased *cryaba* expression thereby impairing lysosomal function by increasing its pH and inhibiting enucleation and organelle degradation within the lens fiber cells (Cui et al., 2020; Gao et al., 2017).

Further studies have shown that αB-crystallins are also involved in cholesterol biosynthesis within the lens (Frankfater et al., 2020; Park et al., 2023). Cholesterols are synthesized within the lens and are necessary at high concentrations to maintain the physical properties of the lens fiber cell membranes and reduce oxygen transport to decrease oxidative damage (Zelenka, 1984; Widomska and Subczynski, 2019). Defects in cholesterol synthesis are associated with cataracts (Cenedella, 1996). In zebrafish, cataract formation in *cryaba* −/− null mutants were reversed by increasing cholesterol biosynthesis (Park et al., 2023). In this study by Park et al., the *cryaba* −/− was crossed with a *nrf2 −/−* mutant, which surprisingly rescued the lens abnormalities. Transcriptome analysis further delineated that the double mutants (*cryaba −/−*, *nrf2 −/−*) showed upregulation of enzymes involved in cholesterol biosynthesis including *cyp51*, *dhcr24*, *ebp*, *las*, *msmol*, *sqlea*, *hmgcra*, and *hmgcs1.80 Nrf2* encodes a transcription factor that mitigates oxidative stress, and so it is not clear as to the exact mechanism by which this resulted in increased cholesterol biosynthesis. Further, this study did not show specific downregulation of these cholesterol enzymes in the *cryaba −/−* mutants, however, mouse studies with the *Cryab* R120G knock-in did show that the mutant protein decreased the content of some minor sterols within the lens (Frankfater et al., 2020). Thus, additional studies are needed for better understanding the relationship between the αB-crystallins and cholesterol synthesis within the lens.

While the α-crystallins are typically associated with congenital cataracts, variants in *CRYAB* are also associated with skeletal myopathies and cardiomyopathies, including myofibrillar, restrictive, and hypertrophic subtypes (Dimauro et al., 2018; Thorkelsson and Chin, 2024). Within the heart, CRYAB prevents protein misfolding and protects against proteotoxicity (Mitra et al., 2013; McLendon and Robbins, 2015). Variants in *CRYAB* impair chaperone function thereby preventing translocation from the cytosol to the cytoskeleton, hindering oligomer exchange and decreasing binding of substrates. This ultimately leads to abnormal muscle striations and tissue dysfunction, causing many cardiovascular diseases (Thorkelsson and Chin, 2024). In zebrafish embryos, while *cryaa* is predominantly expressed in the developing lens, *cryaba* and *cryabb* are expressed in other cells including muscle progenitor cells and the primordial heart by 48 hpf (Farnsworth et al., 2021). In Mishra et al., both the *cryaba −/−* and the *cryabb −/−* mutants had normal hearts, but showed pericardial edema and dilated cardiomyopathy in response to environmental stress or exposure to external glucocorticoids (Mishra et al., 2018). The G154S variant in *CRYAB*, associated with myofibrillar myopathy, was evaluated in zebrafish and resulted in myofibrillar aggregates and disruption of muscle fiber structure (Reilich et al., 2010; Vattemi et al., 2011; Cannone et al., 2023). In addition the double *cryaba −/−; nrf2 −/−* zebrafish mutant showed worse cardiac findings especially with treatment of glucocorticoids than either of the single mutants, suggesting a link between oxidative stress and αB-crystallins within the heart (Park et al., 2023). However, the *cryabb* gene was increased in *nrf2 −/−* mutant heart tissues as well as by exogenous treatment with dexamethasone, suggesting a differential response in gene regulation as well as oxidative stress of the two *cryab* genes in zebrafish. Thus, additional studies are needed to determine the different roles of *cryaba* and *cryabb* in the zebrafish heart, as well as how this information could be translated to mammalian systems with only 1 C*ryab* gene.

### ß-crystallins and γ-crystallins

There is greater divergence of the ß- and γ-Crystallin genes between fish, mice, and humans, and thus less information is available as to their function in the lens during development as well as post-natally. Single cell transcriptome analysis recently showed that the four *cryba1* (*cryba1l1*, *cryba1l2*, *cryba1a*, *cryba1b*), two *cryba2* (*cryba2a*, *cryba2b*), one *cryba4*, four *crybb1* (*crybb1*, *crybb1l1*, *crybb1l2*, *crybb1l3*) and one *crybb2* genes were predominantly expressed in the lens fiber and/or lens epithelial cells in zebrafish embryos. The *crybb3* gene is unique in that it is not expressed within the lens during zebrafish development and shares overlapping expression with the *crybg* genes (*crybg1a*, *crybg1b*) in the pharyngeal and otic regions. The remaining *crybg* genes, *crybg2* and *crybgx* have indeterminate expression and lens fiber expression, respectively (Farnsworth et al., 2021). To date there are no models for any of the endogenous zebrafish ß- or γ-Crystallin genes. However, zebrafish have been used to analyze the human *CRYBB1* promoter, showing that lens-specific expression was driven by a150 bp region around the start site that contained a binding site for the MAF transcription factor (Hou et al., 2006).

The majority of the γ-Crystallins in zebrafish are in the M-group, which evolved for an aqueous environment. Single cell transcriptome analysis demonstrated the majority of *crygm* genes are expressed within lens fiber cells, although there are some expressed in skeletal muscle, gastrointestinal epithelial cells, kidney and liver (Farnsworth et al., 2021). As knockout analysis of the endogenous γ-Crystallins would likely yield minimal information regarding human disease, these models have not been reported. Nevertheless, the I4F and V76D *CRYGD* variants predicted to destabilize the crystallin protein have been transgenically expressed in zebrafish under the *cryaa* promotor (Mishra et al., 2012; Wu et al., 2016). While 30%–60% of zebrafish carrying either of the variants showed mild lens abnormalities (crystal-like droplets), fish that had the double I4F/V76D transgene had the greatest penetrance (∼80%) of lens defects with more than half having significant abnormalities (irregular protuberances in the center of the lens). These lens defects showed similarity to the *cryaa*−/− null mutant published by the same group as well as the R49C *CRYAA* and R120G *CRYAB* transgenic zebrafish (Mishra et al., 2018; Wu et al., 2018). Thus, although the γD-Crystallin does not have a zebrafish equivalent, these studies showed that knock-in of variants in *CRYGD* shares a phenotype with the zebrafish and human α-Crystallin genes.

## Conclusion

Variants in the Crystallin genes account for approximately 1/3 of inherited cases of childhood cataracts. Although the α-Crystallins are well-characterized small heat shock proteins that act as chaperones to prevent protein aggregation, the ß- and γ-Crystallins are a separate superfamily with less defined functions. Genetic testing of individuals with congenital cataracts has yielded a plethora of pathogenic variants in all of the Crystallin genes, but their specific functions during lens development are not well understood (Bell et al., 2020; Berry et al., 2020). Zebrafish studies have given important insight into the role of the α-Crystallin genes in lens development and determining downstream targets and signaling pathways. Although there is greater species divergence of the ß- and γ-Crystallins than α-Crystallins, the use of zebrafish to study the effects of pathogenic crystallin variants remains an important tool for better understanding the pathogenesis of congenital cataracts.

Ongoing studies are curating the *CRY* gene variants associated with cataracts in humans. As demonstrated by previous studies, zebrafish can be used to study the pathogenic effects of these variants by using lens-specific Cre transgenic lines as well as CRISPR-Cas9 gene manipulation. Improving our understanding of congenital cataract pathogenesis can ultimately decrease childhood vision loss by deploying molecular targeted treatments to prenatally preventing or postnatally curing the molecular abnormalities.
